# The Risk of Developing Cervical Cancer in the Elderly: Who Benefits From Screening After the Age of 60?

**DOI:** 10.1097/LGT.0000000000000893

**Published:** 2025-04-18

**Authors:** Renée M.F. Ebisch, Celine Buijssen, Nicole C.M. Visser, Albert G. Siebers, Ruud L.M. Bekkers

**Affiliations:** 1 Radboud University Medical Center, Department of Obstetrics and Gynaecology, Nijmegen, The Netherlands; 2 Catharina Hospital, Department of Obstetrics and Gynaecology, Eindhoven, The Netherlands; 3 Eurofins PAMM, Department of Pathology, Eindhoven, The Netherlands; 4 Palga, Houten, The Netherlands; 5 GROW School for Oncology and Reproduction, Maastricht University, Maastricht, The Netherlands

**Keywords:** cervical cancer, screening, cervical intraepithelial neoplasia, high-risk human papillomavirus, elderly, high-risk human papillomavirus

## Abstract

**Objectives:**

Population-based cervical cancer screening in the Netherlands ends at age 60. This retrospective cohort study aims to identify a subgroup of people over 60 years who are at increased cervical cancer risk, and may benefit from extended screening.

**Methods:**

People with a cervix, aged 59–61 with an abnormal exit smear (index smear), conducted as part of the screening program between 2000 and 2004, were identified from the Dutch nationwide pathology databank. A 1:3 matching was obtained with people without an abnormal screening smear at the same age. Incidence rate ratios (IRR) were calculated for the risk of developing cervical cancer or cervical intraepithelial neoplasia (CIN) later in life. Up to 22 years of follow-up was obtained.

**Results:**

A total of 10,368 people were identified. The IRR for CIN and cervical cancer was increased for people with an abnormal index smear. This risk was highest for people with a high-grade index smear, compared with a normal index smear; IRR of high-grade CIN of 104.05 (95% CI = 38.18–353.18) and IRR for cervical cancer of 18.58 (95% CI = 5.31–61.07). The majority (82%) of people with an abnormal index test showed normal cytology or histology preceding their CIN or cervical cancer.

**Conclusions:**

People with a cervix with abnormal cytology in their exit screening smear 59–61 years showed a 19 times increased lifelong risk of cervical cancer and more than 100 times increased risk for CIN. Because this increased risk was not limited to a specific timeframe, prolonged screening or adjusted diagnostic follow-up for this specific group should be considered.

Between 2015 and 2020, a total of 4,790 Dutch people were diagnosed with cervical cancer.^1^ The incidence of cervical cancer diagnoses was, as expected, highest in people aged 30–44 years old, because of screening initiation, and the average time needed for a persistent high-risk human papillomavirus (hrHPV) infection to develop cervical cancer. However, in that same time period, 1,248 people (26%) diagnosed with cervical cancer were older than 60 years.^1^

People with a cervix in the Dutch nationwide cervical cancer screening program are invited for a cervical smear (liquid-based cytology) every 5–10 years from the age 30 until the age of 60, leaving those above the age of 60 without regular cervical screening opportunities.^2^ The Dutch cancer registry shows similar revised European standardized rates for cervical cancer for people of 45–59 years of age, and people of 60 years and above.^1^ In many high-income countries, even a bipolar pattern with a first top on the incidence curve around the age of 35–40 years and a second top around the age of 65–80 years can be seen.^3^ This second top on the incidence curve has been hypothesized to be associated with reactivation of a latent hrHPV infection because of degeneration of the immune system with age.^4^ It could also be explained by midlife change of sexual partners, or it is a reflection of the screening history of the birth cohort caused by insufficient screening history,^5,6^ or with challenges associated with diagnostic follow-up due to an invisible transformation zone in postmenopausal changes of the cervix.^7^ Indeed, a Danish study showed an average age of nonscreened people with cervical cancer to be 76 years, which could be in line with the second top on the incidence curve.^5^ Previous studies have shown that even after adjusting for treatment disparities, cancer-specific mortality is higher in the elderly,^8,9^ and in recent decades the relative 5-year survival rate of cervical cancer increased in all age groups, except for people above 75 years. Indeed the mortality of people with cervical cancer above the age of 75 is more than 2 times higher than other age groups.^1^

This study aimed to identify people over 60 years of age with an increased risk for developing cervical cancer by their cervical cancer exit screening test (in this study further identified as index smear), in order to offer extended screening to a selected group of people with a cervix to decrease incidence and mortality of cervical cancer.

## METHODS

This population-based retrospective comparative cohort study included people with a cervix who participated in the last screening round, at age 60, of the Dutch population-based cervical cancer screening program. People with an abnormal exit smear result, defined as atypical squamous cells of undetermined significance (ASC-US) or worse, taken within the cervical screening program at age 59–61 between January 1, 2000 and December 31, 2004 were identified. A control group was obtained by 3:1 frequency matching based on age, year of inclusion, and urbanization grade, with people registered in the same period with cervical exit smears without abnormal cells, defined as negative for intraepithelial lesion or malignancy (NILM). These smears are labeled as the index screening smear. Patients were identified based on a pseudonymized combination of date of birth and maiden name. The database was subsequently coded using anonymized patient numbers. For all identified people, the complete history of histology and cytopathology results of the cervix were retrieved up to August 5, 2022, resulting in a maximum of 22 years of follow-up. The most severe follow-up diagnosis was identified as endpoint, where histology was given priority over cytology results.

For reporting of cervical smears, The Dutch CISOE-A classification system, which can easily be translated into the Bethesda nomenclature, was used.^10^ People with an abnormal index smear, including ASC-US, atypical glandular cells (AGC), low-grade squamous intraepithelial lesion (LSIL), atypical squamous cells, cannot exclude a high-grade lesion (ASC-H), and high-grade squamous intraepithelial lesion (HSIL), were identified from the Dutch nationwide pathology databank (Palga; Houten, Netherlands). Next, index cytology as well as follow-up cytology were categorized as high-grade (HSIL and ASC-H) and low-grade (ASC-US and LSIL). Histology samples were classified as low-grade cervical intraepithelial neoplasia (CIN), grade 1 (CIN1), and high-grade CIN (CIN2 and CIN3). Only histological results with a clear pathological description of invasive disease were classified as cancer. The group with no CIN during follow-up included people with normal histology or cytology results, or people without cytology or histology follow-up results after their index smear, indicating that no cervical premalignancy or malignancy has been diagnosed. To account for prevalent hrHPV-related CIN and cervical cancer, abnormal Pap smears and CIN lesions were censored for 2 years after inclusion because these indicate immediate follow-up after the index smear. People with cervical cancer or a hysterectomy within 2 years were excluded from analysis. Follow-up for people undergoing a hysterectomy after 2 years was censored from the date of the hysterectomy and follow-up for people diagnosed with cervical cancer after 2 years was censored from the date of the diagnosis because some were not treated surgically but received chemoradiation. The general follow-up protocol after an abnormal Pap smear in the Netherlands in this period was a colposcopy, either or not combined with a biopsy or loop excision of the transformation zone, depending on the assessment of the colposcopist, followed by follow-up for at least 12 months depending on the cytology and hrHPV result. In the Netherlands, treatment of CIN2 and CIN3 is advised for postmenopausal women and see-and-treat loop excision of the transformation zone is offered in case of high-grade cytology and a high-grade impression during colposcopy.

All statistical analyses were performed with SPSS version 29. Numbers and percentages of all cervical cancer and CIN were calculated per inclusion group and combined groups. The high-grade CIN group includes high-grade histology and cytology results, and low-grade CIN includes low-grade histology and cytology. Furthermore, range of interval in days, between the index screening smear and highest follow-up diagnosis was calculated for each cervical cancer or CIN detected, taking censoring as described above into account. Cervical intraepithelial neoplasia and cancer were labeled as “new” if a normal histology or cytology result followed after the index smear and preceded the worst result, otherwise they were labeled as persistent.

Person-years were calculated until the first diagnosis of cervical cancer or the most severe follow-up diagnosis. Incidence rates (IR) and Incidence rate ratios (IRR) with 95% CIs of cervical cancer and CIN between the index screen test grouped as normal cytology, low-grade cytology, and high-grade cytology were calculated. The analysis does not account for death or immigration because these are expected to be similar in both groups, therefore not affecting the IRR.

The study was approved by the scientific committee of Palga (study approval no.: lzv2022–87). The study was exempt from institutional review board approval because data were gathered retrospectively and analyzed anonymously.

## RESULTS

**FIGURE 1:**
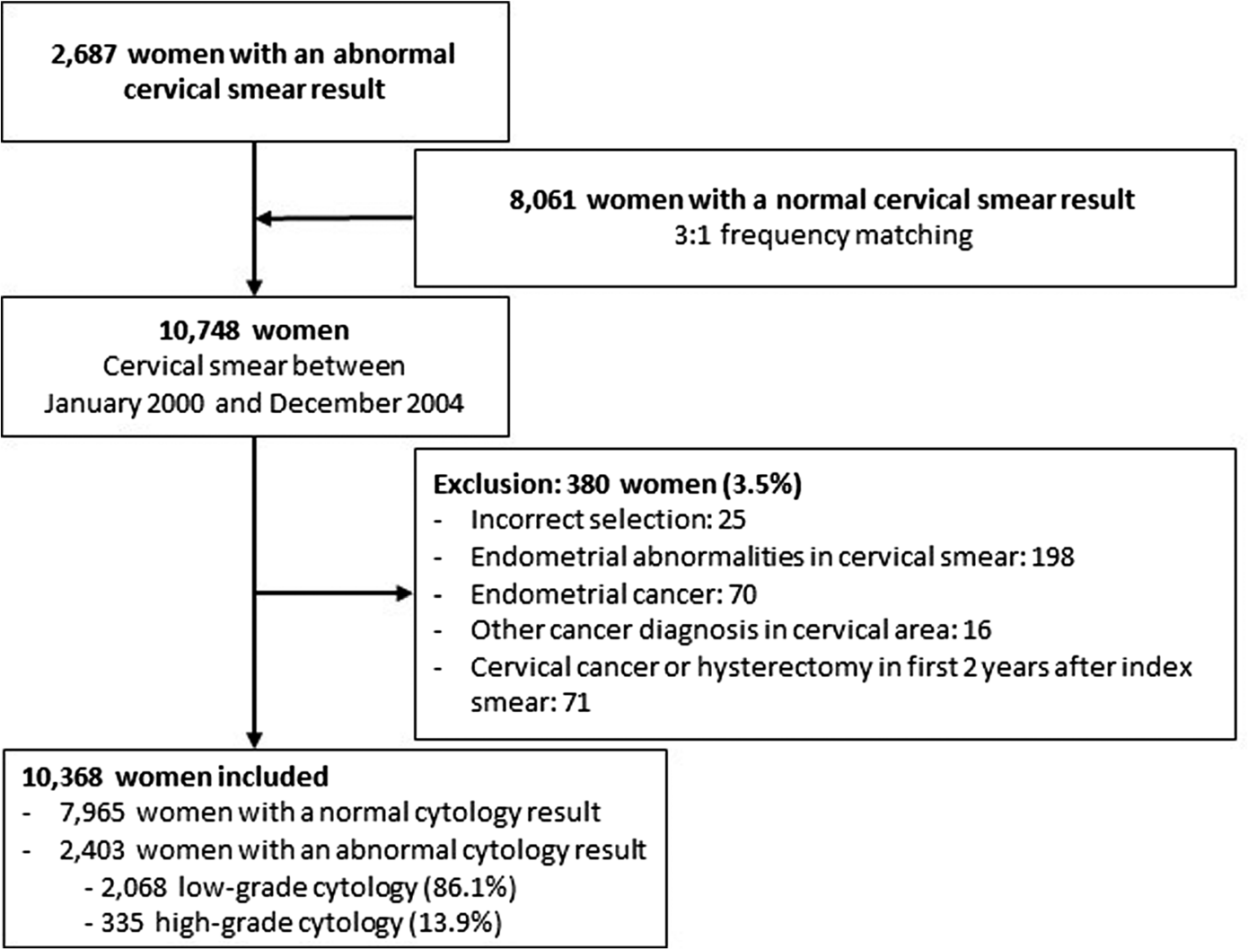
Flowchart of the study population.

We identified 2,687 people with an abnormal index smear, which was taken at age 59–61, between January 1, 2000, and December 31, 2004 (case group). We matched them with 8,061 people with a normal cervical smear, which was taken at age 59–61 during the same period in time (control group). In total, 10,748 people were initially selected, and a total of 380 people were excluded, of which 284 were in the case group and 96 in the control group, resulting in a total of 10,368 people included in the study. The majority of exclusions were related to endometrial abnormalities that were detected by the cervical smear (Figure 1).

The study database consisted of 208.570 person-years of follow-up, of which 160,412 years were in the control group and 48,158 years in the cases group. Of all included people, a total of 21 (0.2%) developed cervical cancer, 47 (0.4%) people developed high-grade CIN, and 161 (1.6%) people developed low-grade CIN. The group with an NILM index smear (normal cytology) showed the lowest risk of developing abnormalities after more than 2 years because only 0.1% of people developed cervical cancer, 0.1% high-grade CIN, and 0.5% developed low-grade CIN. People who were included with a low-grade index smear developed cervical cancer in 0.4%, high-grade CIN in 1.0% and low-grade CIN in 4.6% after more than 2 years. The highest percentage of cervical cancer and CIN was found in the high-grade cytology group, with 6.3% people with high-grade CIN and 1.8% people with cervical cancer (Table 1).

**TABLE 1:** Number of People With Cervical cancer and CIN in Relation to Their Index Smear

	Results of the index smear at age 59–61			
	Normal cytology (%)	Low-grade cytology (%)	High-grade cytology (%)	Total (%)
No CIN	7,908 (99.3)	1,944 (94.0)	287 (85.6)	10,139 (97.8)
Low-grade	44 (0.5)	96 (4.6)	21 (6.3)	161 (1.6)
*Low-grade cytology*	*39*	*71*	*15*	*125*
*Low-grade histology*	*5*	*25*	*6*	*36*
High-grade	5 (0.1)	21 (1.0)	21 (6.3)	47 (0.4)
*High-grade cytology*	*2*	*4*	*1*	*7*
*High-grade histology*	*3*	*17*	*20*	*40*
Cervical cancer	8 (0.1)	7 (0.4)	6 (1.8)	21 (0.2)
Total	7,965	2,068	335	10,368

CIN indicates cervical intraepithelial neoplasia.

The majority of abnormalities in people with an abnormal index smear and abnormal cytology or histology result during follow-up, developed after a preceding normal cytology or histology result. In total, 82% of all abnormalities were new cytological abnormalities, and only 18% resulted after a persistent cytological abnormality (Table 2).

**TABLE 2:** Number of Persistent or New Abnormalities in Relation to Their Index Smear

	Results of the index smear at age 59–61		
	Low-grade cytology	High-grade cytology	Total
Low-grade			
*Persistent (%)*	*16 (17%)*	*3 (14%)*	*19 (16%)*
*New (%)*	*80 (83%)*	*18 (86%)*	*98 (84%)*
High-grade			
*Persistent (%)*	*3 (14%)*	*8 (38%)*	*11 (26%)*
*New (%)*	*18 (86%)*	*13 (62%)*	*31 (74%)*
Cervical cancer		1	5
*Persistent (%)*	*0 (0%)*	*0 (0%)*	*0 (0%)*
*New (%)*	*4 (100%)*	*1 (100%)*	*5 (100%)*
Total			
*Persistent (%)*	*19 (16%)*	*11 (26%)*	*30 (18%)*
*New (%)*	*102 (84%)*	*32 (74%)*	*134 (82%)*

**FIGURE 2:**
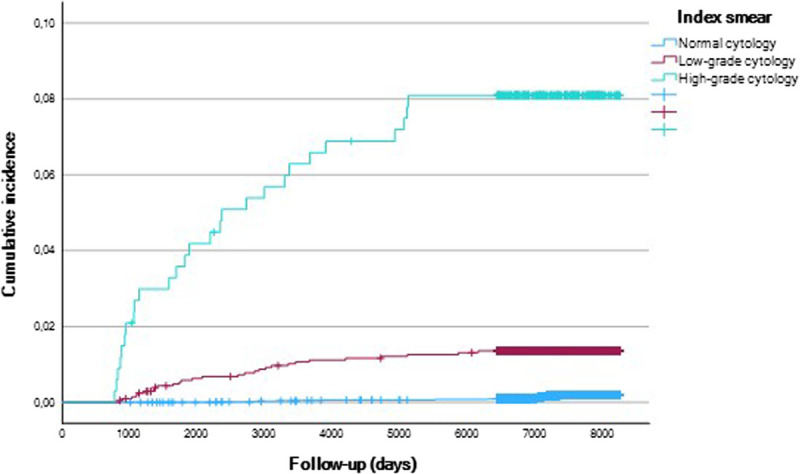
Kaplan-Meier curve estimating the cumulative incidence of high-grade cervical intraepithelial neoplasia (CIN) and cervical cancer in relation to their index cervical smear.

It was not feasible to predict when people in a certain group would develop cervical high-grade CIN or cervical cancer, given the small numbers. Also, high-grade abnormalities and cervical cancer were diagnosed throughout the entire follow-up period. The lowest number of high-grade CIN and cervical cancer was found closely following a normal index smear (Figure 2).

The overall IRR for developing high-grade CIN and cervical cancer is significantly increased for people with an abnormal index smear at cervical screening exit, when compared with a normal cervical smear with IRR of 27.98 (95% CI = 11.09–90.61) and 5.41 (95% CI = 2.08–15.04), respectively. The IRR for high-grade CIN and cervical cancer was highest in people in the high-grade index group, compared with people in the normal index group with an IRR for high-grade CIN of 104.05 (95% CI = 38.18–353.18) and IRR for cervical cancer of 18.58 (95% CI = 5.31–61.07). For the low-grade index group compared with the normal cytology group, the IRRs were also significantly increased with an IRR 16.6 (95% CI = 5.93–54.86) for high-grade CIN and 3.37 (95% CI = 1.04–10.63) for cervical cancer.

People in the high-grade cytology group were 19 times more likely to develop cervical cancer compared with the normal cytology group, and 5 times more likely compared with the low-grade cytology group (Table 3).

**TABLE 3:** Estimated Incidence Rates and Incidence Rate Ratios Per 1,000 Person-Years, for Cervical cancer and Low-Grade and High-Grade CIN, in Relation to the Index Smear After More Than 2 Years Follow-Up

Estimated incidence rate ratios between normal index cytology and abnormal index cytology.					
	Abnormal index cytology48,158 person-years		Normal index cytology160,412 person-years		
Follow-up outcome	Observed no	IR	Observed no	IR	IRR (95% CI)
No CIN	2,231	46.33	7,908	49.30	0.94 (0.90–0.99)
Low-grade	117	2.43	44	0.27	8.86 (6.21–12.83)
High-grade	42	0.87	5	0.03	27.98 (11.09–90.61)
Cervical cancer	13	0.27	8	0.05	5.41 (2.08–15.07)
Total no	2,403		7,965		
**Estimated incidence rate ratios between normal index cytology and high-grade index cytology.**					
	High-grade index cytology 6,475 person-years		Normal index cytology 160,412 person-years		
Follow-up outcome	Observed no	IR	Observed no	IR	IRR (95% CI)
No CIN	287	44.32	7,908	49.30	0.90 (0.80–1.01)
Low-grade	21	3.24	44	0.27	11.82 (6.68–20.32)
High-grade	21	3.24	5	0.03	104.05 (38.18–353.18)
Cervical cancer	6	0.93	8	0.05	18.58 (5.31–61.07)
**Total no**	335		7,965		
**Estimated incidence rate ratios between normal index cytology and low-grade index cytology.**					
	Low-grade index cytology 41,683 person-years		Normal index cytology 160,412 person-years		
Follow-up outcome	Observed no	IR	Observed no	IR	IRR (95% CI)
No CIN	1,944	46.64	7,908	49.30	0.95 (0.90–0.99)
Low-grade	96	2.30	44	0.27	8.40 (5.82–12.29)
High-grade	21	0.50	5	0.03	16.16 (5.93–54.86)
Cervical cancer	7	0.17	8	0.05	3.37 (1.04–10.63)
**Total no**	2,068		7,965		

IR indicates incidence rate; IRR, incidence rate ratio; Observed no, observed number; total no, total number.

## DISCUSSION

This Dutch population-based cohort study, which included 10,748 people with a cervix, showed that people with an abnormal cervical smear result at age 59–61 (exit of screening age) are at increased risk of developing cervical cancer and high-grade CIN at an older age, compared with people who have a normal cervical smear result at the same age. The majority of CIN and cervical cancer in the group of people with an abnormal index smear was preceded by a normal cytology or histology result, indicating that regular follow-up for only 2 years with a normal cervical cytology does not reduce their risk similar to that of people with a normal screening exit smear at 60 years old.

Our results are similar to those of Wang et al, who studied cervical screening with cytology at age 61–65 and showed that this was associated with a statistically significant reduction of subsequent cervical cancer risk for people who were unscreened, or screened with abnormalities, in their 50s. In people screened with normal results in their 50s, the risk for future cancer was not sizeable, and the risk reduction associated with continued screening appeared limited.^11^

Regular screening participation has been shown to reduce cervical cancer incidence by 82%, and mortality caused by cervical cancer by 92% for people who participate in screening.^12,13^ The American Cancer Society, the American College of Obstetricians and Gynecologists, and the US Preventive Services Task Force recommend screening cessation at 65 years old with 10 prior years of normal screening. The NHS cervical screening program in the United Kingdom screens people up to 64 years. The Dutch screening program only screens people up to 60 years old. It can be debated if this age should be adjusted to 65 or even up to 70 years of age, or whether identification and screening of specific risk groups above a certain age is more sensible.^14^

Comparing different studies, from different countries and time periods, is challenging because of the global differences and time-related changes in cervical cancer screening.^15^ A recent Danish study has shown that of all people with cervical cancer, 18.2% had been sufficiently screened and were eligible to stop screening according to the IARC recommendations,^5^ and about 40% of people with a cervix in the United Kingdom developing cervical cancer at age 65 or over had been screened regularly and were discharged from the program with normal cytology results.^16^ Different European studies have reported that 25%–40% of people with cervical cancer have never been screened before their diagnosis. Castanon et al. showed that the risk of developing cervical cancer at older age is related to the previous screening participation and screening results, with the highest risk for people with an abnormal screening result at ages 50–59 and no test at 60–64. They suggest that the upper age of screening should depend on previous screening participation and results.^17^

In the Netherlands, women can exit the screening program when tested hrHPV negative at the age of 60. However, current Dutch guidelines include the advice to perform follow-up screening after 5 years, and up to 10 years after treating cervical abnormalities after the age of 55. The recent introduction of the more sensitive hrHPV test for screening purposes decreases the chance of CIN in the following years compared with cytology screening;^18^ however, people aged >60 may not have been offered this test. The HPV-prevalence data from Northern Europe covering ages up to 65+ years have indicated a moderate rebound after menopause in people with normal cytology.^19,20^ A few people will acquire a new hrHPV infection after the age of 65, but it is suggested that most cervical cancers in older people are the result of infections acquired many years previously because a persisting risk is associated with early age at first intercourse.^21^ Although latent hrHPV infections sometimes lead to precancerous changes late in life, a substantial proportion of older people who are hrHPV positive may already have subclinical precancerous cells.^22^

In a Danish population, regular screening is offered to people with a cervix up to 64 years with an HPV check-out test at age 60–64. Catch-up hrHPV testing was offered to people with a cervix aged 69 and above. It showed a 30.2% participation rate with an average age of 74.6 years. An overall hrHPV positivity rate of 4% was found, which shows no sign of rebound hrHPV prevalence after menopause and it did not reflect the relatively high cervical cancer incidence in the elderly, possibly caused by selection bias because of the limited participation rate.^3^ Also, Tranberg et al showed that catch-up HPV testing is feasible because a higher number of CIN2+ was detected in people with a cervix who were offered an hrHPV test at age 65–69, possibly preventing the development of cervical cancer.^23^ Because half the cervical cancer deaths in England occur in people with a cervix aged over 65 years. In order to decrease the number of cervical cancer deaths in this group of elderly patients, UK experts advise to perform studies on self-sampling in the elderly up to at least 80 years, to determine whether self-sampling is a feasible and cost-effective option for hrHPV testing.^14^

A strength of this study is the population-based design and the embedment of the study into the routine Dutch screening program, thus providing evidence on the real-life performance of cytologic screening. Furthermore, this study consists of a long follow-up period ranging between 17 and 22 years. Limitations are the retrospective nature of the study and that results on hrHPV status could not have been included, as well as data on screening history and medical history of patients, tobacco use, and immunocompromised status was not available. Unfortunately, long-term follow-up results with hrHPV testing in people over 60 years old are not available yet. It previously has been reported that up to 50% of high-grade CIN lesions might be missed when only cervical biopsies are taken compared to a diagnostic loop excision of the transformation zone in postmenopausal people with a cervix. This could indicate an even larger risk for people with an abnormal cervical smear.^7^ Because we identified 82% new abnormalities during follow-up, this could include previously undetected abnormalities, which may indicate an overestimation of new abnormalities. Also, patients without cytology or histology follow-up results were presumed not have a cervical malignancy; however, this may result in some missed cases because it may also be an indicator of lack of follow-up.

In our normal index cytology group at age 60 years, about 1 in 1,000 people developed cervical cancer or high-grade CIN during long-term follow-up. This implies that a normal cervical smear result at the age of 60 results in a very low risk of developing cervical cancer later in life, and people are therefore less in need of an adjusted long-term follow-up program. With the high sensitivity of hrHPV-based screening, this risk is expected to be even lower in people with a negative hrHPV test at age 60 in the current Dutch screening program. Additionally, the low number of abnormalities found in this group can be found at any time interval after the negative cytology test, indicating that continuing screening after the age of 60 years may not be (cost)efficient for people with a normal cytology (and/or hrHPV negative) result at 60 years.

## CONCLUSION

This population-based study shows an increased long-life risk of cervical hrHPV-related cervical cancer and CIN in people with an abnormal exit cytological screening test at 59–61. Adjusted diagnostic follow-up, HPV catch-up testing, and prolonged, or even lifelong, screening for this specific group should be considered depending on the availability of resources and depending on the different health care systems worldwide, and at the same time keeping benefit/harm ratio in mind.
